# Tobacco control policies and smoking cessation treatment utilization: A moderated mediation analysis

**DOI:** 10.1371/journal.pone.0241512

**Published:** 2021-08-30

**Authors:** Johannes Thrul, Kira E. Riehm, Joanna E. Cohen, G. Caleb Alexander, Jon S. Vernick, Ramin Mojtabai

**Affiliations:** 1 Department of Mental Health, Bloomberg School of Public Health, Johns Hopkins University, Baltimore, Maryland, United States of America; 2 Sidney Kimmel Comprehensive Cancer Center at Johns Hopkins, Baltimore, Maryland, United States of America; 3 Centre for Alcohol Policy Research, La Trobe University, Melbourne, Australia; 4 Department of Health, Behavior and Society, Bloomberg School of Public Health, Johns Hopkins University, Baltimore, Maryland, United States of America; 5 Institute for Global Tobacco Control, Bloomberg School of Public Health, Johns Hopkins University, Baltimore, Maryland, United States of America; 6 Center for Drug Safety and Effectiveness, Bloomberg School of Public Health, Johns Hopkins University, Baltimore, Maryland, United States of America; 7 Department of Epidemiology, Bloomberg School of Public Health, Johns Hopkins University, Baltimore, Maryland, United States of America; 8 Division of General Internal Medicine, Johns Hopkins Medicine, Baltimore, Maryland, United States of America; 9 Department of Health Policy and Management, Bloomberg School of Public Health, Johns Hopkins University, Baltimore, Maryland, United States of America; University of California San Francisco (retired), UNITED STATES

## Abstract

**Background:**

Tobacco policies, including clean indoor air laws and cigarette taxes, increase smoking cessation in part by stimulating the use of cessation treatments. We explored whether the associations between tobacco policies and treatment use varies across sociodemographic groups.

**Methods:**

We used data from 62,165 U.S. adult participants in the 2003 and 2010/11 Tobacco Use Supplement to the Current Population Survey (TUS-CPS) who reported smoking cigarettes during the past-year. We built on prior structural equation models used to quantify the degree to which smoking cessation treatment use (prescription medications, nicotine replacement therapy, counseling/support groups, quitlines, and internet resources) mediated the association between clean indoor air laws, cigarette excise taxes, and recent smoking cessation. In the current study, we added selected moderators to each model to investigate whether associations between tobacco polices and smoking cessation treatment use varied by sex, race/ethnicity, education, income, and health insurance status.

**Results:**

Associations between clean indoor air laws and the use of prescription medication and nicotine replacement therapies varied significantly between racial/ethnic, age, and education groups in 2003. However, none of these moderation effects remained significant in 2010/11. Higher cigarette excise taxes in 2010/2011 were associated with higher odds of using counseling among older adults and higher odds of using prescription medications among younger adults. No other moderator reached statistical significance. Smoking cessation treatments did not mediate the effect of taxes on smoking cessation in 2003 and were not included in these analyses.

**Conclusions:**

Sociodemographic differences in associations between clean indoor air laws and smoking cessation treatment use have decreased from 2003 to 2010/11. In most cases, policies appear to stimulate smoking cessation treatment use similarly across varied sociodemographic groups.

## Introduction

Cigarette smoking is the single largest health risk behavior contributing to morbidity and mortality in the U.S. and responsible for more than 480,000 deaths each year [1]. While quit attempts have increased and smoking prevalence has generally decreased over recent decades, there are entrenched differences in smoking prevalence among population subgroups [2]. Members of racial/ethnic minority groups [2,3] and adults of low socioeconomic status (SES) [2,4] smoke cigarettes at higher rates than their counterparts. For example, 21.3% of adults with an annual household income <$35k smoke, compared to 13.7% of adults in the general US population [2]. Moreover, research has consistently shown differences in smoking prevalence by health insurance availability [5–7]. Current smoking prevalence among people whose health care is government funded due to lower income (Medicaid beneficiaries; 23.9%) is more than twice that of privately insured individuals (10.5%) [2]. Lack of private insurance may also limit access to medical care, magnifying the contribution of smoking to substantial health inequities, including marked disparities in cancer incidence, mortality, and cardiovascular disease risk in vulnerable populations [8,9]. Better understanding of how to reduce smoking disparities is an urgent public health priority [10].

Tobacco control policies, including cigarette excise taxes and clean indoor air laws, have helped to reduce rates of cigarette smoking in the US [11–13]. However, these policies vary substantially on a state and local level [14–16]. As of March 2021, there are 633 local jurisdictions in the US with their own cigarette tax rates, with wide ranges from $1.25 to $7.16 per pack [16]. Comprehensive clean indoor air laws also vary on a local level, especially for communities in states that lack comprehensive statewide laws [14]. Research investigating the role of tobacco control policies in smoking cessation thus needs to account for local variation in these policies.

Moreover, not all population subgroups may benefit equally from tobacco control policies. Despite the enactment of these policies, the quit attempts of smokers of low socioeconomic status (SES) are less successful, although they are just as likely to try quitting as other smokers [1,17]. Many low-SES smokers report past negative experiences with quitting [18] and have internalized smoking stigma, which may be associated with reduced self-efficacy for quitting [19]. Additional obstacles to quitting faced by low-SES smokers include stronger nicotine dependence [4], social networks comprised of smokers, and strong pro-smoking social norms [20,21].

Increasing use of evidence-based smoking cessation treatments (e.g., Nicotine Replacement Therapy (NRT), prescription medication, counseling, telephone quit lines, etc.) among all sociodemographic groups of smokers is a key strategy to reduce smoking prevalence on a population level [22,23], especially if there are synergies between implementation of tobacco control policies, such that these policies drive the uptake of evidence-based smoking cessation treatment use. However, the utilization of smoking cessation treatments is unevenly distributed across the US population. Indeed, analyses of Tobacco Use Supplement to the Current Population Survey (TUS-CPS) data showed that smoking cessation attempts unaided by evidence-based strategies were more likely among men, young adults, Blacks, and individuals with lower income [24]. Recent analyses of the Population Assessment of Tobacco and Health (PATH) study also confirmed that adults who identified as Black, Hispanic, or other race/ethnicity report less use of pharmacotherapy for quitting smoking [25] and Black adults are less likely to attain successful short- or long-term smoking cessation compared to Whites [26].

We previously used the TUS-CPS data to demonstrate that U.S. smoking cessation increased from 2003 to 2010/11 and that changes in cigarette taxes and clean indoor air laws accounted for a substantial proportion of this increase [27]. Moreover, we found that cessation treatment use partly mediated the association of clean indoor air laws and smoking cessation in 2003 and the association of cigarette excise taxes and smoking cessation in 2010/11 [28]. In the current study, we extend this work by examining whether the mediating effect of cessation treatments is similar across men and women, different age groups, racial/ethnic groups, and income levels, as well as groups with different health insurance coverage. Given that reducing smoking prevalence among vulnerable subpopulations is an urgent public health priority, this is a question of foremost importance for tobacco control research.

## Methods

### Sample

The TUS-CPS is a national population-level study of tobacco use conducted at regular intervals in conjunction with CPS. We used data from the 2003 and the 2010 and 2011 waves of the TUS-CPS. For 2003, the supplement was administered in February, June, and November 2003; for 2010 and 2011, TUS was administered in May and August 2010, and in January 2011. CPS uses a multi-stage stratified sampling procedure to interview a nationally representative sample of the non-institutionalized civilian U.S. population aged 15 years and older in 2003 and 18 years and older in 2010 and 2011. Approximately 64% of respondents complete the TUS-CPS by telephone and 36% in person. Most interviewees reported on their own tobacco use behavior; 20% reported as proxies for other household members. Additional information regarding the TUS-CPS can be found by visiting the TUS-CPS website [29].

We limited our sample to past-year adult smokers, aged 18 and older, who reported on their own smoking behavior in TUS-CPS. A total of 34,842 participants in 2003 and 27,323 in 2010 and 2011 (for simplicity referred to as the 2011 TUS-CPS henceforth) met these criteria and were included.

Analyses are based on publicly available de-identified data that were exempt from review by the Johns Hopkins Bloomberg School of Public Health Institutional Review Board.

### Measures

*Past-year smoker* status was ascertained by asking participants about their smoking pattern exactly 12 months before the interview. This question was asked separately from current every-day and someday smokers as well as those who had quit in the past-year. Individuals who reported smoking every day or on some days one year ago were rated as past-year smokers for this study.

*Quitting in the past-year* was ascertained by responses “not at all” to the question “Do you now smoke cigarettes every day, some days, or not at all?” among past-year smokers. Among people who responded “not at all”, 82.7% had last smoked 30 days or longer before the time of interview.

*Cigarette excise taxes* and state and local *clean indoor air laws* were ascertained for each participant at the time point exactly one year before the time of their TUS-CPS interview. This timeframe was chosen because questions about smoking behavior in the TUS-CPS covered the past-year. To reflect the variation in tobacco control policies at the state and local level, we obtained data on state and local cigarette excise taxes and clean indoor air laws from the American Nonsmokers’ Rights Foundation (ANRF). Total excise tax was computed as the sum of federal, state, and local taxes. ANRF ascertains data on state and local clean indoor air laws separately for laws affecting workplace areas, bars, and restaurants. We used the ANRF categorization of these laws into those imposing a “100% smoke free policy,” a “qualified 100% smoke free policy,” laws providing “some” coverage, and “no coverage”. In situations where the state and local laws affecting a participant were inconsistent, we chose the more comprehensive law. While some states pre-empt, or disallow, local tobacco control laws, the number of such pre-emptive state laws affecting clean indoor air policies decreased over the study period: 12 states had such laws in 2010, down from 18 in 2000 [30]. State and local law data were linked to the TUS-CPS data using state and county FIPS codes. Because state and local laws affecting workplace, bars, and restaurants are strongly correlated (r range = .61 to.83), an average clean indoor law index was computed.

*Smoking cessation treatment use* was assessed by asking past-year smokers who had quit or had made an attempt to quit about the methods they had used. These methods included nicotine replacement treatments (nicotine gum, lozenge, patch, inhaler, or nasal spray), prescription medications (Zyban, Wellbutrin, or bupropion; Chantix or varenicline was added in 2010/11), one-on-one counseling, stop smoking clinic, class, or support group (combined into a “counseling/groups”), telephone help line or quit line, and the internet.

*Family income* and *health insurance coverage* were assessed in the 2003, 2010 and 2011 Annual Social and Economic (ASEC) Supplement of CPS that is administered in March of each year and has partial overlap with the TUS-CPS sample [31]. As such, for a smaller proportion of TUS-CPS participants information on income and health insurance is available. For this study, annual family income was categorized into three categories (<20,000$, 20,000$—<75,000$, and ≥75,000$) and health insurance into 3 mutually exclusive groups: public insurance (Medicare, Medicaid, VA/CHAMPS, Indian Health Services), private insurance (either through job or personally obtained) with or without public insurance; and no health insurance.

In addition to questions about smoking and smoking cessation treatments, TUS-CPS also collected sociodemographic data including sex, age (18–29, 30–49, 50–64, 65+), race/ethnicity (non-Hispanic White, non-Hispanic Black, Hispanic, other), education (< High School, High School graduate or GED, some college but not bachelor’s degree, bachelor’s degree or higher), employment status (employed, unemployed, not in labor force), and marital status (married or living as married, widowed, divorced or separated, never married). We also adjusted the analyses for country region and state-level expenditure for tobacco prevention programs compared to US Centers for Disease Control and Prevention (CDC) recommended expenditure for years 2003 and 2011 compiled by the Campaign for Tobacco-Free Kids [32].

### Analyses

We analyzed the data in two stages. First, we examined variations in the use of different smoking cessation treatments across sociodemographic and health insurance groups using contingency tables.

**Fig 1:**
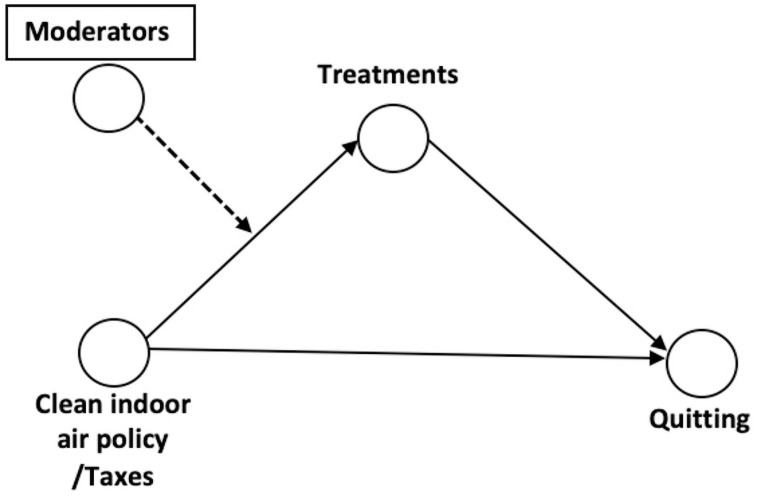
Moderated mediation model. The use of smoking cessation treatments mediates the association between tobacco control policies (clean indoor air policy/taxes) and smoking cessation. Sociodemographic differences among past-year smokers moderate this mediation effect. Analytically, the models in the current manuscript tested whether the association between tobacco control policies and the use of smoking cessation treatments varied by sociodemographic group.

In a previous publication, we investigated how the use of smoking cessation treatments mediated the association between clean indoor air laws and taxes, and smoking cessation [28]. In the current study, we conducted moderated mediation analyses (Fig 1) using structural equation modeling with binary outcomes and multiple binary mediators [33,34]. These analyses investigate whether the relationship between a predictor (e.g., tobacco control policies) and a mediator (smoking cessation treatment use), varies across sociodemographic subgroups of smokers (moderator). For example, higher cigarette taxes (predictor) may incentivize smokers to use smoking cessation treatments (mediator) to attain smoking cessation (outcome). Yet, the extent to which different smokers utilize smoking cessation treatments may be impacted by sociodemographic characteristics (moderator), such that, for example, low-income smokers are potentially less likely to use NRT or cessation medications because of the associated costs. In all these models, it is assumed that the average effect of use of smoking cessation treatments (mediator) on smoking cessation (outcome) remains constant across sociodemographic subgroups. An introduction to moderated mediation analyses can be found elsewhere [35]. Because both the mediators (use of different smoking cessation treatments) and most of the moderating factors were categorical, we conducted these moderated mediation analyses by multi-group structural equation modeling. All coefficients in these models were fixed to be constant across groups except for the coefficients linking the exposure (clean indoor air laws in 2003; clean indoor air laws or taxes in 2010/11) to each mediator. These latter coefficients were allowed to vary across groups [36]. Moderated effects were tested by comparing these coefficients for each treatment across groups. To avoid spurious findings, moderation was tested for preselected sociodemographic variables that were associated with smoking cessation treatment use in previous research, including sex, race/ethnicity, age, education, income and health insurance. These models also adjusted for employment status, marital status, country region, and state-level expenditure for tobacco prevention programs compared to CDC recommendations. All analyses were conducted separately for the 2003 period and the 2010/11 period. These analyses build on our previous set of analyses that examined whether and to what extent cessation treatment policies mediated the effect of clean indoor air laws and cigarette excise taxes on recent smoking cessation [28]. Analyses for 2003 were limited to the effect of clean indoor air laws because only these laws were associated with smoking cessation in 2003. Analyses for 2010/11 included both clean indoor air laws and cigarette excise taxes, as cessation treatments mediated the effect of both [28].

As our interest was to examine variations across sociodemographic groups in the effects of clean indoor air laws and excise taxes on the use of various treatments, in the main analyses we constrained the coefficients linking the mediators with smoking cessation to be equal across sociodemographic groups. In sensitivity analyses we relaxed this constraint and allowed the coefficients linking mediators with smoking cessation to vary across groups.

Analyses were conducted using Stata 16.0 software (StataCorp LLC, College Station, TX, 2019). Structural equation modeling analyses were conducted using the *gsem* routine of Stata which accommodates binary outcomes and mediators as well complex survey data with *successive difference replications* as required for analyses of census data [37]. Survey and replicate weights were included in all analyses to compute population representative estimates and confidence intervals. All percentages reported are weighted. Due to the high number of comparisons conducted, a conservative p<0.01 cutoff was used for deciding the statistical significance of the tests.

## Results

### Sample characteristics

We previously described characteristics of past-year smokers in the 2003 and the 2010/11 samples [27,28]. Briefly, the majority of participants in both time periods were male (53.7% in 2003 and 54.1% in 2010/11), non-Hispanic White (75.7% and 74.4%), and employed (66.0% and 58.6%). The average age of the participants was 41.41 years (standard error [SE] = .05) in 2003 and 42.69 years (SE = 0.10) in 2010/11. The proportion married or living as married were 43.7% in 2003 and 39.9% in 2010/11. The South region had the largest proportion of participants in both set of samples (37.5% in 2003 and 39.3% in 2010/11), and the Northeast region had the smallest proportion (18.0% and 16.4%, respectively).

### Tobacco control policies

We have previously reported on variations in clean indoor air laws and taxes in the two time periods [27,28]. State and local governments varied considerably in their adoption of tobacco control policies and the extent of coverage changed markedly over time. In 2003, only 1.9% of past-year smokers lived in states and localities with 100% smoke-free workplace laws, 8.2% in states and localities with 100% smoke-free bar clean indoor air laws and 9.0% in states and localities with 100% smoke-free restaurant laws. These numbers increased to 47.7%, 44.3% and 53.5%, respectively, in 2010/11. Excise taxes also increased over time from an average of $1.00 (SE = .001) to $2.25 (SE = .005). The proportion of past-year smokers who quit did not change much over two periods: 7.3% in 2003 and 7.8% in 2010/11 quitted smoking. The most commonly used treatments in both 2003 and 2010/11 were nicotine replacement therapies, used by 10.3% of past-year smokers in 2003 and 2010/11, followed by prescription medications (3.9% in 2003, 5.9% in 2010/11), counseling/groups (1.3% in 2003, 1.7% in 2010/11), quitlines (0.5% in 2003, 1.3% in 2010/11), and the Internet (0.6% in 2003, 0.8% in 2010/11) (Tables 1 and 2).

**Table 1:** Use of smoking cessation treatments and smoking cessation among 34,842 past-year smokers in the Tobacco Use Supplement to the Current Population Survey, 2003, according to sociodemographic characteristics and type of treatment.

Groups	N	Quit smoking N (%)	Prescription medications^a^	NRT^b^	Counseling/Groups^c^	Quitline	Internet
			N (%)	N (%)	N (%)	N (%)	N (%)
Total	34842	—	1508 (3.9)	3877 (10.3)	518 (1.3)	233 (0.5)	217 (0.6)
Total quit smoking	--	2488 (7.3)	215 (14.0)	519 (13.8)	90 (16.3)	31 (12.3)	28 (12.2)
Sex							
Male	16792	1168 (6.8)	581 (3.1)	1731 (9.5)	197 (1.0)	74 (0.4)	78 (0.5)
Female	18050	1320 (7.8)	927 (4.8)	2146 (11.2)	321 (1.6)	159 (0.7)	139 (0.8)
Comparison	--	--	X^2^_df = 1_ = 184.01, p<0.001	X^2^_df = 1_ = 84.00, p<0.001	X^2^_df = 1_ = 99.34, p<0.001	X^2^_df = 1_ = 63.86, p<0.001	X^2^_df = 1_ = 48.24, p<0.001
Race/ethnicity							
Non-Hispanic White	27862	2033 (7.4)	1339 (4.5)	3311 (11.2)	427 (1.4)	186 (0.5)	186 (0.7)
Non-Hispanic Black	2879	151 (5.5)	55 (1.8)	220 (7.1)	37 (1.1)	20 (0.7)	10 (0.3)
Hispanic	2180	171 (8.1)	32 (1.2)	158 (6.5)	17 (0.6)	11 (0.5)	10 (0.5)
Other	1921	133 (7.5)	82 (3.4)	188 (9.2)	37 (1.5)	16 (0.6)	11 (0.5)
Comparison	--	--	X^2^_df = 3_ = 387.92, p<0.001	X^2^_df = 3_ = 257.51, p<0.001	X^2^_df = 3_ = 44.87, p<0.001	X^2^_df = 3_ = 8.33, p = 0.040	X^2^_df = 3_ = 24.65, p<0.001
Age, years							
18–29	7348	735 (10.0)	174 (2.0)	576 (7.0)	56 (0.6)	56 (0.6)	52 (0.7)
30–49	16050	1002 (6.3)	772 (4.3)	1916 (11.1)	229 (1.2)	103 (0.4)	111 (0.7)
50–64	8172	492 (5.9)	469 (5.8)	1089 (13.1)	176 (1.9)	53 (0.6)	50 (0.7)
65+	2913	205 (7.3)	89 (3.1)	272 (9.1)	43 (1.6)	18 (0.7)	4 (0.2)
Comparison	--	--	X^2^_df = 3_ = 397.06, p<0.001	X^2^_df = 3_ = 427.88, p<0.001	X^2^_df = 3_ = 248.42, p<0.001	X^2^_df = 3_ = 14.18, p = 0.003	X^2^_df = 3_ = 14.29, p = 0.003
Education							
<HS gradate	6403	369 (6.1)	181 (2.5)	570 (7.8)	69 (0.9)	38 (0.5)	11 (0.1)
HS graduate or GED	13947	892 (6.4)	563 (3.6)	1402 (9.3)	168 (1.0)	74 (0.4)	48 (0.3)
College, < bachelor’s degree	10089	783 (8.1)	540 (5.0)	1280 (12.0)	193 (1.7)	94 (0.8)	98 (1.0)
Bachelor’s degree or more	4403	444 (10.1)	224 (4.8)	625 (13.3)	88 (1.7)	27 (0.5)	60 (1.4)
Comparison	--	--	X^2^_df = 3_ = 208.29, p<0.001	X^2^_df = 3_ = 389.00, p<0.001	X^2^_df = 3_ = 91.47, p<0.001	X^2^_df = 3_ = 52.83, p<0.001	X^2^_df = 3_ = 267.57, p<0.001
Employment							
Employed	22822	1713 (7.6)	999 (3.9)	2563 (10.3)	290 (1.1)	137 (0.4)	158 (0.7)
Unemployed	2480	142 (5.7)	83 (2.8)	230 (8.6)	38 (0.9)	16 (0.5)	15 (0.7)
Not in labor force	9540	633 (6.8)	426 (4.2)	1084 (10.6)	190 (1.8)	80 (0.7)	44 (0.4)
Comparison	--	--	X^2^_df = 2_ = 29.58, p<0.001	X^2^_df = 2_ = 23.07, p<0.001	X^2^_df = 2_ = 86.17, p<0.001	X^2^_df = 2_ = 33.40, p<0.001	X^2^_df = 2_ = 28.38, p<0.001
Marital status							
Married/living as married	15854	1193 (7.7)	818 (4.8)	1943 (11.5)	259 (1.4)	107 (0.5)	99 (0.6)
Widowed	1806	103 (5.7)	72 (3.8)	178 (9.1)	24 (1.3)	8 (0.4)	6 (0.4)
Divorced/separated	8015	441 (5.3)	385 (4.6)	966 (11.9)	123 (1.3)	63 (0.6)	53 (0.7)
Never married	9167	751 (8.2)	233 (2.2)	790 (7.6)	112 (1.0)	55 (0.5)	59 (0.7)
Comparison	--	--	X^2^_df = 3_ = 255.55, p<0.001	X^2^_df = 3_ = 272.45, p<0.001	X^2^_df = 3_ = 27.95, p<0.001	X^2^_df = 3_ = 5.78, p = 0.123	X^2^_df = 3_ = 7.32, p = 0.062
Family income^d^							
<$20K	4774	285 (6.3)	154 (2.7)	435 (8.4)	62 (1.0)	38 (0.6)	22 (0.5)
$20-<$75K	7748	633 (8.0)	404 (4.6)	944 (11.3)	120 (1.3)	53 (0.6)	45 (0.5)
$75K+	1896	191 (10.5)	114 (5.5)	270 (13.7)	30 (1.6)	8 (0.3)	24 (1.5)
Comparison	--	--	X^2^_df = 2_ = 156.16, p<0.001	X^2^_df = 2_ = 224.84, p<0.001	X^2^_df = 2_ = 14.41, p<0.001	X^2^_df = 2_ = 7.58, p = 0.023	X^2^_df = 2_ = 73.41, p<0.001
Health insurance^d^							
Private	8205	724 (8.9)	459 (5.1)	1052 (12.1)	126 (1.4)	55 (0.5)	64 (0.8)
Public	2563	136 (5.4)	104 (3.9)	284 (11.1)	57 (2.0)	29 (1.1)	13 (0.6)
None	2870	174 (6.3)	77 (2.1)	223 (6.5)	25 (0.6)	12 (0.2)	8 (0.2)
Comparison	--		X^2^_df = 2_ = 149.62, p<0.001	X^2^_df = 2_ = 252.03, p<0.001	X^2^_df = 2_ = 61.12, p<0.001	X^2^_df = 2_ = 46.16, p<0.001	X^2^_df = 2_ = 28.45, p<0.001

Note: All percentages are weighted.

^a^Includes Zyban, Wellbutrin, or bupropion; Chantix or varenicline was added in 2010/11.

^b^NRT = Nicotine replacement therapies. Includes nicotine gum, lozenge, patch, inhaler, or nasal spray.

^c^Includes one-on-one counseling, stop smoking clinic, class, or support group.

^d^Family income and health insurance coverage were assessed in the Annual Social and Economic (ASEC) Supplement of CPS administered in March wave of the CPS and has partial overlap with the TUS-CPS sample. As such, this information is not available for the full TUS-CPS sample.

**Table 2:** Use of smoking cessation treatments and smoking cessation among 27,323 past-year smokers in the Tobacco Use Supplement to the Current Population Survey, 2010/11, according to sociodemographic characteristics and type of treatment.

Groups	N	Quit smoking N (%)	Prescription medications^a^	NRT^b^	Counseling/Groups^c^	Quitline	Internet
			N (%)	N (%)	N (%)	N (%)	N (%)
Total	27323	--	1866 (5.9)	3066 (10.3)	555 (1.7)	482 (1.3)	236 (0.8)
Total quit smoking	--	2114 (7.8)	292 (16.0)	447 (14.6)	103 (18.6)	71 (14.2)	42 (17.6)
Sex							
Male	13538	1046 (7.6)	745 (4.6)	1414 (9.4)	215 (1.4)	168 (1.0)	88 (0.6)
Female	13785	1068 (8.0)	1121 (7.4)	1652 (11.3)	340 (2.0)	314 (1.7)	148 (1.1)
Comparison	--	--	X^2^_df = 1_ = 123.11, p<0.001	X^2^_df = 1_ = 31.56, p<0.001	X^2^_df = 1_ = 16.48, p<0.001	X^2^_df = 1_ = 44.38, p<0.001	X^2^_df = 1_ = 19.04, p<0.001
Race/ethnicity							
Non-Hispanic White	21263	1677 (7.9)	1629 (6.8)	2512 (10.9)	419 (1.7)	395 (1.4)	200 (0.9)
Non-Hispanic Black	2587	161 (6.6)	97 (3.2)	237 (9.0)	76 (2.6)	39 (1.1)	10 (0.4)
Hispanic	1857	153 (8.2)	50 (2.4)	143 (6.8)	22 (1.0)	23 (1.1)	11 (0.5)
Other	1616	123 (8.1)	90 (5.1)	174 (10.6)	38 (1.4)	25 (1.2)	15 (1.2)
Comparison	--	--	X^2^_df = 3_ = 118.96, p<0.001	X^2^_df = 3_ = 44.79, p<0.001	X^2^_df = 3_ = 27.50, p<0.001	X^2^_df = 3_ = 2.59, p = 0.460	X^2^_df = 3_ = 10.37, p = 0.016
Age, years							
18–29	5306	562 (10.2)	157 (2.5)	457 (7.6)	74 (1.0)	87 (1.0)	64 (1.2)
30–49	11241	823 (7.2)	827 (6.6)	1315 (11.1)	214 (1.7)	201 (1.4)	96 (0.7)
50–64	8080	526 (6.4)	679 (7.6)	1001 (11.6)	217 (2.4)	158 (1.7)	63 (0.7)
65+	2696	203 (7.4)	203 (7.1)	293 (10.3)	50 (1.5)	36 (0.9)	13 (0.5)
Comparison	--	--	X^2^_df = 3_ = 143.90, p<0.001	X^2^_df = 3_ = 68.37, p<0.001	X^2^_df = 3_ = 66.09, p<0.001	X^2^_df = 3_ = 21.13, p<0.001	X^2^_df = 3_ = 18.51, p<0.001
Education							
<HS gradate	4428	248 (5.2)	227 (4.2)	423 (8.2)	90 (1.5)	67 (1.1)	21 (0.4)
HS graduate or GED	10920	742 (7.1)	698 (5.6)	1120 (9.5)	185 (1.4)	163 (1.0)	54 (0.5)
College, < bachelor’s degree	8538	737 (8.6)	710 (7.1)	1077 (11.7)	203 (2.0)	192 (1.8)	102 (1.3)
Bachelor’s degree or more	3437	387 (11.5)	231 (6.2)	446 (12.2)	77 (1.9)	60 (1.5)	59 (1.5)
Comparison	--	--	X^2^_df = 3_ = 68.98, p<0.001	X^2^_df = 3_ = 57.76, p<0.001	X^2^_df = 3_ = 13.71, p = 0.003	X^2^_df = 3_ = 25.36, p<0.001	X^2^_df = 3_ = 56.11, p<0.001
Employment							
Employed	15906	1260 (7.9)	1045 (5.6)	1724 (10.1)	268 (1.4)	246 (1.1)	142 (0.8)
Unemployed	2908	192 (7.1)	162 (4.8)	295 (8.6)	49 (1.3)	57 (1.4)	30 (1.0)
Not in labor force	8509	662 (7.8)	659 (6.8)	1047 (11.4)	238 (2.4)	179 (1.6)	64 (0.7)
Comparison	--	--	X^2^_df = 2_ = 25.28, p<0.001	X^2^_df = 2_ = 22.26, p<0.001	X^2^_df = 2_ = 47.29, p<0.001	X^2^_df = 2_ = 10.58, p = 0.005	X^2^_df = 2_ = 2.85, p = 0.240
Marital status							
Married/living as married	11380	917 (8.0)	954 (7.6)	1303 (10.7)	228 (1.7)	181 (1.3)	107 (0.9)
Widowed	1527	113 (7.3)	123 (6.9)	196 (12.2)	39 (2.7)	35 (2.0)	8 (0.4)
Divorced/Separated	6649	440 (6.7)	493 (6.6)	809 (11.4)	164 (2.1)	148 (1.7)	51 (0.7)
Never married	7767	644 (8.3)	296 (3.1)	758 (8.6)	124 (1.2)	118 (1.0)	70 (0.9)
Comparison	--	--	X^2^_df = 3_ = 214.87, p<0.001	X^2^_df = 3_ = 54.04, p<0.001	X^2^_df = 3_ = 32.19, p<0.001	X^2^_df = 3_ = 17.14, p<0.001	X^2^_df = 3_ = 4.11, p = 0.250
Family income^d^							
<$20K	7717	499 (6.4)	438 (4.8)	869 (9.8)	194 (1.9)	179 (1.7)	48 (0.6)
$20-<$75K	15216	1182 (8.0)	1057 (5.9)	1672 (10.3)	294 (1.6)	249 (1.2)	130 (0.8)
$75K+	4390	433 (9.4)	371 (7.8)	525 (11.0)	67 (1.4)	54 (1.1)	58 (1.3)
Comparison	--	--	X^2^_df = 2_ = 62.70 p<0.001	X^2^_df = 2_ = 4.94, p = 0.085	X^2^_df = 2_ = 4.42, p = 0.110	X^2^_df = 2_ = 15.66, p<0.001	X^2^_df = 2_ = 15.44, p<0.001
Health insurance^d^							
Private	5786	524 (9.1)	473 (7.2)	674 (11.1)	115 (1.7)	98 (1.4)	56 (0.8)
Public	2854	208 (7.3)	220 (6.7)	361 (12.4)	77 (2.6)	70 (2.1)	27 (1.0)
None	2814	198 (7.1)	91 (2.7)	251 (8.2)	35 (0.8)	34 (0.9)	17 (0.6)
Comparison	--	--	X^2^_df = 2_ = 68.98, p<0.001	X^2^_df = 2_ = 31.92, p<0.001	X^2^_df = 2_ = 36.86, p<0.001	X^2^_df = 2_ = 16.00, p<0.001	X^2^_df = 2_ = 3.12, p = 0.210

Note: All percentages are weighted.

^a^Includes Zyban, Wellbutrin, or bupropion; Chantix or varenicline was added in 2010/11.

^b^NRT = Nicotine replacement therapies. Includes nicotine gum, lozenge, patch, inhaler, or nasal spray.

^c^Includes one-on-one counseling, stop smoking clinic, class, or support group.

^d^Family income and health insurance coverage were assessed in the Annual Social and Economic (ASEC) Supplement of CPS administered in March wave of the CPS and has partial overlap with the TUS-CPS sample. As such, this information is not available for the full TUS-CPS sample.

### Variations in the use of smoking cessation services across population groups

There were significant sociodemographic variations in the use of smoking cessation treatments among past-year smokers. Women were consistently more likely to use all forms of treatment both in 2003 and 2010/11. Similarly, non-Hispanic Whites were more likely to use most forms of treatment in both periods, with the few exceptions of use of quitlines in both 2003 and 2010/11, use of internet in 2010/11, as well as use of counseling and groups by “other” racial/ethnic groups in 2003 and non-Hispanic Blacks in 2010/11.

There were some consistencies in the patterns of use of services among past-year smokers according to age as well. Compared to other age groups, adults in the 50–64 years age range were most likely and those in the 18–29 years age group least likely to use prescription medications, replacement therapies and counseling and groups in both 2003 and 2010/11 periods.

Past-year smokers with higher education were more likely than those with less education in both periods to use all services, a gradient in use of services according to family income was also found for prescription medications and replacement therapies in both 2003 and 2010/11 and for counseling and groups in 2003: individuals with family income <$20,000 were least likely to use these services; whereas, those with family incomes ≥ $75,000 were most likely. Past-year smokers with no health insurance coverage, compared to those with private and public insurance, were less likely to use any kind of treatment in both periods, including treatments that are typically free of charge or not reimbursed by health insurance, such as internet resources and quitlines. The associations with marital status were less consistent.

### Moderated mediation analyses

There were variations in mediated effects across sociodemographic groups in both 2003 and 2010/11, although some of the mediation coefficients did not reach a statistically significant level due to small number of past-year smokers in some demographic groups who used smoking cessation treatments (e.g., racial/ethnic minorities). All effects represent associations between tobacco control policies (predictor) and smoking cessation treatment use (mediator) and assume that the average effect of the mediator on smoking cessation (outcome) remains constant across sociodemographic subgroups.

More stringent clean indoor air laws were associated with increased odds of prescription medication and nicotine replacement therapy use among past-year smokers identifying as non-Hispanic White and non-Hispanic Black in 2003. Furthermore, more stringent clean indoor air laws were associated with increased odds of prescription medication and nicotine replacement therapy use in the age groups 50–64 and 30–49 in 2003 (Table 3). However, such racial/ethnic and age variations were not apparent in 2010/11. Associations between clean indoor air laws and prescription medication use in 2003 were somewhat smaller among past-year smokers who had not graduated high school and those with advanced degrees. A similar pattern was noted with regard to higher education and use of nicotine replacement therapies. However, moderation effects by education were not apparent in 2010/11.

**Table 3:** Mediating effect of smoking cessation treatments in the association of clean indoor air policies and smoking cessation in different sociodemographic groups of past-year smokers (moderated mediation), 2003 and 2010/11^a^.

Group	2003 survey		2010/11 survey	
	Prescription medications^b^	NRT^c^	Prescription medications^b^	NRT^c^
Overall	1.08 (1.02–1.13)	1.11 (1.07–1.15)	1.08 (1.03–1.14)	1.09 (1.05–1.13)
Sex				
Male	1.09 (1.00–1.19)	1.07 (1.02–1.13)	1.06 (0.98–1.14)	1.05 (0.99–1.12)
Female	1.08 (1.00–1.16)	1.04 (0.99–1.09)	1.06 (1.00–1.13)	1.01 (0.96–1.06)
Comparison of groups	X^2^_df = 1_ = 0.06, p = 0.812	X^2^_df = 1_ = 1.05, p = 0.306	X^2^_df = 1_ = 0.00, p = 0.973	X^2^_df = 1_ = 2.13, p = 0.145
Race/ethnicity				
Non-Hispanic White	1.14 (1.06–1.22)	1.09 (1.04–1.14)	1.06 (1.00–1.13)	1.02 (0.97–1.06)
Non-Hispanic Black	1.12 (0.87–1.45)	1.28 (1.16–1.42)	1.06 (0.89–1.26)	1.09 (0.96–1.25)
Hispanic	0.61 (0.43–0.85)	0.83 (0.72–0.95)	0.98 (0.72–1.34)	1.15 (0.98–1.35)
Other	0.80 (0.69–0.92)	0.83 (0.73–0.94)	1.06 (0.85–1.32)	1.04 (0.90–1.22)
Comparison of groups	X^2^_df = 3_ = = 22.32, p<0.001	X^2^_df = 3_ = 44.92, p<0.001	X^2^_df = 3_ = 0.26, p = 0.967	X^2^_df = 3_ = 2.68, p = 0.443
Age group				
18–29	0.76 (0.64–0.88)	0.92 (0.85–1.01)	1.09 (0.95–1.26)	0.99 (0.91–1.09)
30–49	1.12 (1.02–1.21)	1.06 (1.01–1.12)	1.06 (0.99–1.15)	1.05 (0.99–1.12
50–64	1.19 (1.08–1.38)	1.15 (1.07–1.23)	1.06 (0.98–1.14)	1.03 (0.97–1.10)
65+	0.97 (0.79–1.20)	0.96 (0.86–1.07)	0.97 (1.19–1.12)	1.02 (0.90–1.15)
Comparison of groups	X^2^_df = 3_ = 34.96, p<0.001	X^2^_df = 3_ = 22.78, p<0.001	X^2^_df = 3_ = 1.94, p = 0.584	X^2^_df = 3_ = 1.24, p = 0.743
Education				
<HS gradate	0.64 (0.54–0.76)	1.07 (0.99–1.17)	1.13 (1.00–1.28)	1.08 (1.00–1.19)
HS graduate or GED	1.25 (1.13–1.36)	1.14 (1.07–1.21)	0.99 (0.92–1.07)	1.02 (0.95–1.08)
College, < bachelor’s degree	1.16 (1.07–1.27)	1.05 (1.00–1.13)	1.13 (1.04–1.21)	1.02 (0.95–1.09)
Bachelor’s degree or more	0.86 (0.75–0.99)	0.90 (0.83–0.98)	1.01 (0.89–1.15)	1.04 (0.94–1.15)
Comparison of groups	X^2^_df = 3_ = 77.67, p<0.001	X^2^_df = 3_ = 23.37, p<0.001	X^2^_df = 3_ = 8.60, p = 0.035	X^2^_df = 3_ = 1.79, p = 0.617
Income^d^				
<$20K	1.06 (0.90–1.25)	1.07 (0.97–1.17)	1.12 (1.01–1.22)	1.03 (0.96–1.12)
$20-<$75K	1.04 (0.93–1.14)	1.05 (0.97–1.14)	1.05 (0.99–1.13)	1.04 (0.98–1.09)
$75K+	1.13 (0.96–1.32)	1.16 (1.05–1.28)	0.99 (0.90–1.09)	1.01 (0.91–1.10)
Comparison of groups	X^2^_df = 2_ = 0.98, p = 0.613	X^2^_df = 2_ = 2.61, p = 0.271	X^2^_df = 2_2.89, p = 0.235	X^2^_df = 2_ = 0.32, p = 0.851
Health insurance^d^				
Private	1.12 (1.01–1.23)	1.14 (1.06–1.22)	0.92 (0.84–1.01)	1.04 (0.95–1.14)
Public	1.13 (0.95–1.35)	1.05 (0.95–1.16)	1.05 (0.91–1.21)	1.04 (0.94–1.16)
None	0.83 (0.58–1.16)	1.01 (0.89–1.14)	1.10 (0.92–1.32)	1.05 (0.93–1.20)
Comparison of groups	X^2^_df = 2_ = 3.66, p = 0.161	X^2^_df = 2_ = 5.31, p = 0.070	X^2^_df = 2_ = 4.37, p = 0.113	X^2^_df = 2_ = 0.02, p = 0.991

Note: Odds Ratios represent associations between clean indoor air policies and use of smoking cessation treatment (mediator) for different sociodemographic groups (moderators).

^a^Coefficients are based on structural equation models adjusting for sex, race/ethnicity, age, education, income, and insurance. In addition, the models adjusted for marital status, employment, country region, and state-level expenditure for tobacco prevention programs compared to CDC recommendations. However, moderating effects were only tested for preselected variables of sex, race/ethnicity, age, education, income, and insurance that were associated with smoking cessation treatment use in past research.

^b^Includes Zyban, Wellbutrin, or bupropion; Chantix or varenicline was added in 2010/11.

^c^NRT = Nicotine replacement therapies. Includes nicotine gum, lozenge, patch, inhaler, or nasal spray.

^d^Family income and health insurance coverage were assessed in the Annual Social and Economic (ASEC) Supplement of CPS administered in the March wave of each CPS and has partial overlap with the TUS-CPS sample. As such, this information is not available for the full TUS-CPS sample.

Associations between clean indoor air laws and the use of prescription medications and nicotine replacement therapies appeared to be somewhat larger among past-year smokers with private or public insurance in both 2003 and 2010/11, although these associations did not reach statistical significance.

Analyses for the association of cigarette taxes with use of smoking cessation treatments in 2010/11 identified few significant moderation effects (Table 4). Associations between cigarette taxes and use of counseling and group therapy appeared be stronger among middle-aged and older past-year smokers; whereas associations between cigarette taxes and use of prescription medications tended to be a stronger among the younger age group. However, this latter association did not reach the predefined p < .01 level of statistical significance (p = 0.011). Other noteworthy, but non-significant moderating effects were also found for health insurance: Associations between taxes and use of both prescription medications and nicotine replacement therapies were stronger among past-year smokers with health insurance; whereas associations between cigarette taxes and use of counseling and group therapy were larger among the uninsured (Table 4).

**Table 4:** Mediating effect of smoking cessation treatments in the association of cigarette excise taxes and smoking cessation in different sociodemographic groups of past-year smokers (moderated mediation), 2010/11^a^.

Group	2010/11 survey		
	Prescription medications^b^	NRT^c^	Counseling/Groups^d^
Overall	1.23 (1.15–1.31)	1.22 (1.15–1.28)	1.19 (1.05–1.34)
Sex			
Male	1.20 (1.08–1.32)	1.21 (1.13–1.30)	1.17 (0.96–1.43)
Female	1.21 (1.11–1.31)	1.16 (1.07–1.26)	1.28 (1.08–1.52)
Comparison of groups	Χ^2^_df = 2_ = 0.01, p = 0.923	Χ^2^_df = 2_ = 0.76, p = 0.383	Χ^2^_df = 2_ = 0.82, p = 0.364
Race/ethnicity			
Non-Hispanic White	1.20 (1.10–1.30)	1.15 (1.07–1.22)	1.25 (1.06–1.48)
Non-Hispanic Black	1.10 (0.88–1.38)	1.36 (1.20–1.55)	1.12 (0.84–1.48)
Hispanic	1.58 (1.16–2.14)	1.35 (1.10–1.63)	1.40 (0.83–2.39)
Other	1.14 (0.76–1.70)	1.21 (0.96–1.54)	1.26 (0.69–2.27)
Comparison of groups	Χ^2^_df = 3_ = 4.12, p = 0.249	Χ^2^_df = 3_ = 7.02, p = 0.071	Χ^2^_df = 3_ = 1.06, p = 0.788
Age group			
18–29	1.54 (1.27–1.86)	1.14 (0.99–1.30)	1.31 (1.01–1.68)
30–49	1.21 (1.07–1.36)	1.23 (1.13–1.34)	0.94 (0.76–1.16)
50–64	1.13 (1.02–1.23)	1.16 (1.06–1.27)	1.48 (1.22–1.79)
65+	1.06 (0.90–1.25)	1.20 (1.03–1.39)	1.62 (1.13–2.29)
Comparison of groups	Χ^2^_df = 3_ = 11.20, p = 0.011	Χ^2^_df = 3_ = 1.52, p = 0.677	Χ^2^_df = 3_ = 14.07, p = 0.003
Education			
<HS gradate	1.35 (1.13–1.63)	1.19 (1.04–1.34)	1.25 (0.96–1.62)
HS graduate or GED	1.10 (0.99–1.22)	1.16 (1.06–1.28)	1.10 (0.86–1.43)
College, < bachelor’s degree	1.23 (1.10–1.38)	1.25 (1.14–1.36)	1.36 (1.12–1.67)
Bachelor’s degree or more	1.25 (1.04–1.49)	1.14 (1.01–1.28)	1.19 (0.91–1.52)
Comparison of groups	Χ^2^_df = 3_ = 5.32, p = 0.150	Χ^2^_df = 3_ = 1.77, p = 0.621	Χ^2^_df = 3_ = 2.16, p = 0.541
Income^e^			
<$20K	1.23 (1.08–1.40)	1.27 (1.16–1.40)	1.21 (0.93–1.57)
$20-<$75K	1.25 (1.14–1.36)	1.14 (1.05–1.23)	1.25 (1.05–1.48)
$75K+	1.05 (0.90–1.21)	1.21 (1.08–1.36)	1.27 (1.01–1.62)
Comparison of groups	Χ^2^_df = 2_ = 5.69, p = 0.058	Χ^2^_df = 2_ = 3.87, p = 0.144	Χ^2^_df = 2_ = 0.13, p = 0.939
Health insurance^e^			
Private	1.35 (1.19–1.55)	1.30 (1.16–1.46)	1.21 (0.91–1.58)
Public	1.42 (1.15–1.77)	1.49 (1.31–1.72)	0.95 (0.68–1.34)
None	1.17 (0.88–1.57)	1.10 (0.91–1.34)	1.54 (1.04–2.29)
Comparison of groups	Χ^2^_df = 2_ = 1.20, p = 0.548	Χ^2^_df = 2_ = 7.93, p = 0.019	Χ^2^_df = 2_ = 3.99 p = 0.136

Note: Odds Ratios represent associations between cigarette excise taxes and use of smoking cessation treatment (mediator) for different sociodemographic groups (moderators).

^a^Coefficients are based on structural equation models adjusting for sex, race/ethnicity, age, education, income, and insurance. In addition, the models adjusted for marital status, employment, country region, and state-level expenditure for tobacco prevention programs compared to CDC recommendations. However, moderating effects were only tested for preselected variables of sex, race/ethnicity, age, education, income, and insurance that were associated with smoking cessation treatment use in past research.

^b^Includes Zyban, Wellbutrin, or bupropion; Chantix or varenicline was added in 2010/11.

^c^NRT = Nicotine replacement therapies. Includes nicotine gum, lozenge, patch, inhaler, or nasal spray.

^d^Includes one-on-one counseling, stop smoking clinic, class, or support group.

^e^Family income and health insurance coverage were assessed in the Annual Social and Economic (ASEC) Supplement of CPS administered in the March wave of the CPS and has partial overlap with the TUS-CPS sample. As such, this information is not available for the full TUS-CPS sample.

Coefficients for the association of clean indoor laws and excise taxes in the sensitivity analyses, in which coefficients linking mediators with smoking cessation were allowed to vary across groups, were identical to the main analyses reported in Tables 3 and 4 with one exception: The model for the moderating effect of race/ethnicity on the association between clean indoor air laws and use of prescription medications in 2003 did not converge after removing the constraint. The model converged after removing the Hispanic group from the model and rerunning analyses separately for each mediator (prescription medications and nicotine replacement therapies). A significant moderating effect of race/ethnicity persisted in the modified model (Χ^2^_df = 2_ = 21.56, p<0.001) investigating prescription medication use. Clean indoor air laws were significantly associated with prescription medication use in non-Hispanic Whites (1.13, 95% CI = 1.05–1.21) and the “Other” racial/ethnic group (0.74, 95% CI = 0.62–0.87), but not in non-Hispanic Blacks (1.12, 95% CI = 0.86–1.45). Furthermore, the assumption of a significant association between smoking cessation treatment use and smoking cessation across sociodemographic groups was supported in these sensitivity analyses, as the majority of coefficients were significantly larger than 1 (Table 5).

**Table 5:** Sensitivity analyses for mediator-outcome association across different sociodemographic groups in models in Tables 3 and 4 with significant variations in mediated effects across groups. In these sensitivity analyses we allowed the coefficients linking mediators with smoking cessation to vary across groups ^a^.

Group	2003 survey		2010/11 survey		
	Prescription medications^b^	NRT^c^	Prescription medications^b^	NRT^c^	Counseling/Groups^d^
**Clean indoor air laws**					
Race/ethnicity					
Non-Hispanic White	2.49 (2.26–2.75)	2.62 (2.46–2.78)			
Non-Hispanic Black	0.84 (0.48–1.47)	2.07 (1.57–2.71)			
Hispanic	--^e^	0.45 (0.27–0.77)			
Other	0.82 (0.52–1.31)	3.27 (2.42–4.42)			
Age group					
18–29	2.57 (2.06–3.21)	1.44 (1.26–1.66)			
30–49	2.06 (1.78–2.39)	2.76 (2.54–3.00)			
50–64	2.28 (1.96–2.65)	2.75 (2.43–3.11)			
65+	3.52 (2.46–5.02)	3.72 (3.12–4.44)			
Education					
<HS gradate	1.56 (1.21–2.18)	1.64 (1.36–1.93)	2.58 (1.60–4.14)		
HS graduate or GED	1.72 (1.43–2.07)	2.29 (2.00–2.61)	2.40 (1.89–3.05)		
College, < bachelor’s degree	1.39 (1.18–1.62)	2.40 (2.10–2.74)	1.73 (1.35–2.21)		
Bachelor’s degree or more	1.81 (1.44–2.26)	2.00 (1.75–2.26)	2.05 (1.43–2.95)		
**Excise taxes**					
Age group					
18–29			1.91 (1.27–2.87)		1.28 (0.95–1.73)
30–49			1.85 (1.48–2.32)		1.93 (1.58–2.36)
50–64			2.06 (1.55–2.74)		2.65 (2.11–3.33)
65+			3.13 (2.08–4.71)		1.92 (1.26–2.94)
Health insurance^f^					
Private				1.64 (1.10–2.47)	
Public				2.16 (1.67–2.79)	
None				1.60 (1.08–2.39)	

Note: Odds Ratios represent associations between smoking cessation treatment use (mediators) and the smoking cessation (outcome) for different sociodemographic groups (moderators).

^a^Coefficients are based on structural equation models adjusting for sex, race/ethnicity, age, education, income, and insurance. In addition, the models adjusted for marital status, employment, country region, and state-level expenditure for tobacco prevention programs compared to CDC recommendations.

^b^Includes Zyban, Wellbutrin, or bupropion; Chantix or varenicline was added in 2010/11.

^c^NRT = Nicotine replacement therapies. Includes nicotine gum, lozenge, patch, inhaler, or nasal spray.

^d^Includes one-on-one counseling, stop smoking clinic, class, or support group.

^e^The model with 4 racial/ethnic groups for prescription medications did not converge. The model converged after excluding the Hispanic group.

^f^Health insurance coverage was assessed in the Annual Social and Economic (ASEC) Supplement of CPS administered in the March wave of each CPS which has partial overlap with the TUS-CPS sample. As such, this information is not available for the full TUS-CPS sample.

## Discussion

We examined whether the mediating effect of smoking cessation treatment use between tobacco policies and smoking cessation varied across different sociodemographic groups of past-year smokers in the 2003 and 2010/11 TUS-CPS. We did this by testing differences in associations between tobacco policies and use of smoking cessation treatments by sociodemographic subgroup. Associations between clean indoor air laws and use of prescription medications and nicotine replacement therapies varied significantly between racial/ethnic, age, and education groups in 2003. However, none of these moderation effects remained significant in 2010/11. The association between cigarette excise taxes and use of smoking cessation counseling was significantly moderated by age group in 2010/11 and the same moderator showed a trend-level significance for prescription medications. No other candidate moderator reached statistical significance.

We found significant differences in smoking cessation treatment use among past-year smokers by sociodemographic background. Consistent with existing research based on PATH data [25], African American and Hispanic smokers were less likely to use evidence-based smoking cessation treatments in both 2003 and 2010/11 and the same was the case for young adult smokers. The low adoption of evidence-based smoking cessation strategies may explain why African American smokers try to quit more frequently than Whites, but are less likely to achieve abstinence [38]. In contrast to other studies, our analyses of TUS-CPS data showed higher treatment use among women and those with higher education, which has not been found in more recent PATH data [25]. Findings of the current study confirm that additional efforts to reach young people and racial/ethnic minorities with evidence based smoking cessation strategies are needed.

Moreover, past-year smokers without health insurance had a lower likelihood to use any type of smoking cessation treatment, including treatments like quitlines and internet resources, which are typically free of charge. This could suggest that in addition to treatment costs, there may be other barriers to treatment access, for example barriers related to knowledge, attitudes, and social norms among uninsured smokers. Moving forward, it will be important to use later waves of the TUS-CPS to investigate the impact of the 2010 Patient Protection and Affordable Care Act (ACA), which required many public and private insurers to cover all FDA approved cessation medications and counseling without insurance barriers [1].

Our findings also demonstrated that sociodemographic moderators of the association between tobacco policies and use of smoking cessation treatments among past-year smokers decreased from 2003 to 2010/11, and this was observed for both cigarette taxes and clean indoor air laws. These results extend earlier findings suggesting that the impact of tobacco control policies on population level smoking cessation is, in part, effected through stimulating the use of evidence-based smoking cessation treatments. This pathway between policies and smoking cessation does not seem to differ in magnitude across various sociodemographic groups in more recent years. Existing research has demonstrated a pro-equity impact of tobacco taxes on smoking disparities by socioeconomic status [39,40]. The evidence for clean indoor air laws in reducing smoking inequities is less clear [40], but some studies suggest that nationwide and comprehensive clean indoor air laws may have a more positive impact on equity compared to regional and voluntary policies [39]. Together with this exsiting literature, our findings suggest that tobacco control policies can contribute to a reduction in tobacco-related health disparities. In order to further reduce these disparities, an emphasis on policies that remove barriers to smoking cessation treatment access among vulnerable populations may be needed. For example, the elimination of co-payments for smoking cessation medication has been associated with an increase in medication use, particularly among low-SES smokers [41].

### Limitations

Our analysis has limitations. In addition to smoking cessation treatment use, as investigated in the current study, other behaviours or attitudes may also act as mediators between tobacco policies and smoking cessation. These other factors were subsumed in the “direct effects” in our mediation analysis and further studies are needed to explore their potential contribution both as mediators as well as moderators of the effect of policies. For example, only a minority of smokers in the general population use evidence-based smoking cessation treatments in quit attempts and most smokers try to quit without assistance [23,25,42], despite the limited success of this method [43]. Moreover, the impact of policies may vary among people with different attitudes towards smoking. Due to the strong correlation between clean indoor air laws and cigarette taxes, we were not able to assess their effects in models including both variables. Data analyzed were from 2003 and 2010/11 and it is unclear if findings hold true today. For example, analyses using the newer data could also investigate the use of electronic cigarettes or heated tobacco products for smoking cessation. Updated analyses including the latest wave of 2018–19 TUS-CPS data as well as updated data on tobacco control policies are warranted moving forward.

### Conclusions

Sociodemographic differences in the effect of clean indoor air laws and excise taxes on smoking cessation treatment use have decreased from 2003 to 2010/11. Although we found some evidence of moderation by sociodemographic characteristics, in most cases, tobacco control policies appear to impact smoking cessation by stimulating smoking cessation treatment use similarly across groups of past-year smokers. Taken together, our findings support the expansion of tobacco control policies, including cigarette taxes and clean indoor air laws, and continued investment in evidence-based smoking cessation treatments, to further reduce smoking in the US population. Such tobacco control efforts appear to be efficacious in reducing smoking among vulnerable groups, including young people, racial/ethnic minorities, and those with low education or income. However, additional efforts are needed to promote smoking cessation in these high priority groups for a pro-equity impact of tobacco control policies and close the gap in smoking cessation rates across population subgroups.
